# Transcriptomic and Physiological Evidence for the Relationship between Unsaturated Fatty Acid and Salt Stress in Peanut

**DOI:** 10.3389/fpls.2018.00007

**Published:** 2018-01-22

**Authors:** Na Sui, Yu Wang, Shanshan Liu, Zhen Yang, Fang Wang, Shubo Wan

**Affiliations:** ^1^Shandong Provincial Key Laboratory of Plant Stress, College of Life Science, Shandong Normal University, Jinan, China; ^2^State Key Laboratory of Crop Biology, Shandong Key Laboratory of Crop Biology, College of Life Sciences, Shandong Agricultural University, Tai'an, China; ^3^Shandong Provincial Key Laboratory of Crop Genetic Improvement, Ecology and Physiology, Shandong Academy of Agricultural Sciences, Jinan, China

**Keywords:** transcriptomic profile, ω-3 fatty acid desaturase, unsaturated fatty acid, salt stress, peanut

## Abstract

Peanut (*Arachis hypogaea* L.) is one of the five major oilseed crops cultivated worldwide. Salt stress is a common adverse condition for the growth of this crop in many countries and regions. In this study, physiological parameters and transcriptome profiles of peanut seedlings exposed to salt stress (250 mM NaCl for 4 days, S4) and recovery for 3 days (when transferred to standard conditions for 3 days, R3) were analyzed to detect genes associated with salt stress and recovery in peanut. We observed that the quantum yield of PSII electron transport (ΦPSII) and the maximal photochemical efficiency of PSII (*F*_v_/*F*_m_) decreased in S4 compared with the control, and increased in R3 compared with those in S4. Seedling fresh weight, dry weight and PSI oxidoreductive activity (Δ*I*/*I*_o_) were inhibited in S4 and did not recover in R3. Superoxide dismutase (SOD) and ascorbate peroxidase (APX) activities decreased in S4 and increased in R3, whereas superoxide anion (${\text{O}}_{2}^{-•}$) and hydrogen peroxide (H_2_O_2_) contents increased in S4 and decreased in R3. Transcriptome analysis revealed 1,742 differentially expressed genes (DEGs) under salt stress and 390 DEGs under recovery. Among these DEGs, two DEGs encoding ω-3 fatty acid desaturase that synthesized linolenic acid (18:3) from linoleic acid (18:2) were down-regulated in S4 and up-regulated in R3. Furthermore, ω-3 fatty acid desaturase activity decreased under salt stress and increased under recovery. Consistent with this result, 18:3 content decreased under salt stress and increased under recovery compared with that under salt treatment. In conclusion, salt stress markedly changed the activity of ω-3 fatty acid desaturase and fatty acid composition. The findings provide novel insights for the improvement of salt tolerance in peanut.

## Introduction

Approximately 1,200 million ha of land are affected by salinity (Wicke et al., 2011). Salt stress is a common abiotic stress that adversely affects plant growth, development, and productivity worldwide (Kayani et al., 1990; Mahajan and Tuteja, 2005; Chen M. et al., 2010; Deng et al., 2015; Song and Wang, 2015). High salinity can affect many important biological processes in plants, including photosynthesis, protein synthesis, energy metabolism, and lipid metabolism (Carillo et al., 2011; Zhou et al., 2016). Constitutive and inducible mechanisms have evolved in plants to resist salt stress. Fatty acid composition is essential as an energy reserve and for maintenance of membrane lipids (Mata-Pérez et al., 2016). Under adverse environmental conditions, cell membranes can sense the stress and initiate reactions to protect cells by adjusting the stress perception and rigidity of the cell structure. Membrane integrity, function, and fluidity are largely affected by the lipid composition and unsaturated fatty acid content in plants (Mikami and Murata, 2003). Unsaturated fatty acids play a crucial role in protecting cell membranes and in maintaining the function of membrane proteins (Deuticke and Haest, 1987; Cooke and Burden, 1990).

Unsaturated fatty acids are synthesized by fatty acid desaturases, which introduce double bonds into the hydrocarbon chains of fatty acids. In addition, fatty acid desaturases play an important role in fatty acid metabolism and maintenance of the biological function of membranes in plant cells (Yadav et al., 1993; Singh et al., 2002; Sui et al., 2017). Previous studies have shown that environmental stresses, such as drought, cold, salt, and heat, can induce changes in fatty acid composition, especially the content of linolenic acid (18:3). Furthermore, increased content of 18:3 is conducive for cold tolerance in many plants (Graham and Patterson, 1982). The ω-6 and ω-3 desaturases are important fatty acid desaturases that play pivotal roles in the biosynthesis of linoleic acid (18:2) and 18:3. Furthermore, 18:2 and 18:3 are important components of plant membranes. FATTY ACID DESATURASE 2 (FAD2) and FAD6 are ω-6 desaturases that synthesize dienoic fatty acids and 18:2 using oleic acid (18:1) in the endoplasmic reticulum (ER) and plastids, respectively. FAD2 and FAD6 are expressed in seedlings of Arabidopsis (*Arabidopsis thaliana*) under salinity stress (Feng et al., 2017). Down-regulated expression of *FATTY ACID ELONGATION 1* (*FAE1*) and *FAD2* modifies the fatty acid profile and accumulated storage compounds in *Brassica napus* seeds (Shi et al., 2017). The ω-3 desaturases comprise FAD3, FAD7, and FAD8, which synthesize 18:3 from 18:2 in the ER (FAD3) and plastids (FAD7 and FAD8), respectively (Gibson et al., 1994). The three genes that encode ω-3 fatty acid desaturases are expressed differentially in different organs and under different conditions (Nishiuchi and Iba, 1998; Schöffl et al., 1999; Yu et al., 2009; Teixeira et al., 2010). Previous studies indicate that *PoleFAD7* and *PoleFAD8* expression is up-regulated in response to wounding and low temperature. Furthermore, total 18:3 content increased in both intact and wounded leaves of plants under chilling stress (Teixeira et al., 2010). Overexpression of *LeFAD7*/*LeFAD3* in tomato plants may increase cold tolerance (Liu et al., 2006; Yu et al., 2009). Compared with wild-type plants, transgenic tobacco overexpressing *Brassica FAD3* or Arabidopsis *FAD8* show increased tolerance to drought and osmotic stress in cultured cells (Zhang et al., 2005). Unsaturated fatty acids also play a key role in improvement of salt tolerance in tomato (Wang et al., 2014). An increase in 18:3 content in Arabidopsis overexpressing the ω-3 fatty acid desaturases FAD3 and FAD7 is indicated to be involved in drought and hypoxia stress signaling (Klinkenberg et al., 2014).

Peanut (*Arachis hypogaea* L.) is one of the five most important oilseed crops cultivated worldwide (Chen Z. B. et al., 2010). However, peanut production is challenged by certain adverse environmental conditions. Especially in China, the widely distributed saline-alkaline land limits the productivity of this crop. Salt and chilling stress can damage the photosystem II (PSII) and photosystem I (PSI) complexes in peanut leaves (Qin et al., 2011). The relationship between salt stress and unsaturated fatty acid content in peanut leaves has not been investigated previously. The genes *FAD2-2, FATTY ACID BIOSYNTHESIS 2* (*FAB2*), *SPHINGOID LCB DESATURASE 1* (*SLD1*), and *FAD6* are differentially expressed during seed development, which suggests the involvement of these genes in seed development. However, few fatty acid desaturases in peanut have been functionally validated (Chi et al., 2011), and the involvement of ω-3 fatty acid desaturases and unsaturated fatty acids in salt tolerance in peanut remains unclear.

In recent years, the development of high-throughput sequencing has greatly facilitated genome-wide gene expression analysis. In the present study, Illumina sequencing technology was used to analyze the transcriptome of peanut seedlings under different salt treatments to identify differentially expressed genes (DEGs) associated with salt tolerance in peanut leaves.

## Materials and methods

### Plant materials and growth conditions

Peanut “Luhua14” seeds of uniform size were used. Plump seeds were first soaked in tap water for 12 h. The imbibed seeds were sown in round plastic pots filled with clean river sand. After germination, the seeds were irrigated with half-strength Hoagland solution until the two-leaf stage was attained. Seedlings at the two-leaf stage were established in hydroponic culture with Hoagland solution in a plastic bucket of 30 cm diameter. Salt treatment was applied after hydroponic culture for 10 days. The NaCl concentration in the nutrient solution was increased stepwise from 50 to 250 mM at the rate of 50 mM, with each concentration applied for 12 h. Control seedlings were not treated with NaCl (CK). Seedlings were treated with 250 mM NaCl for 4 days (S4), then transferred from salt stress to standard (ST) conditions for 4 days (R3). After recovery treatment for 3 days, physiological parameters were measured (Figure S1). Then the seedlings were cultured at 28 ± 5°C (day/night) with light intensity of 600 μmol m^−2^ s^−1^, a 15 h light/9 h dark photoperiod, and 70% relative humidity.

### Measurement of seedling fresh weight and dry weight

Fifteen seedlings from each of the three treatments (five seedlings per replicate) were sampled and the seedling fresh weight (FW) was recorded. The sampled seedlings were dried at 105°C for 15 min and at 70°C for 72 h, and then the seedling dry weight (DW) was recorded.

### Lipid extraction and analysis

Leaves from the peanut seedlings of 15 seedlings from each of the three treatments (five seedlings per replicate) were sampled and frozen immediately in liquid nitrogen. Lipids were extracted in accordance with the method described by Siegenthaler and Eichenberger (1984), followed by separation using two-dimensional thin-layer chromatography (TLC) (Xu and Siegenthaler, 1997). For quantitative analysis, fatty acid methyl esters were prepared after the separation of lipids by TLC. Fatty acid composition was determined by gas chromatography (GC-9A, Shimadzu, Kyoto, Japan) using the method described by Chen et al. (1994).

### Measurement of chlorophyll fluorescence

Chlorophyll fluorescence was measured after treatment with 250 mM NaCl for 4 days (S4) and after recovery for 13 days (R3). Chlorophyll fluorescence was independently measured on five plants with a portable fluorometer (FMS2, Hansatech, King's Lynn, UK) following the method described by Kooten and Snel (1990), and used in a previous study (Sui, 2015). Leaves were kept in the dark for 1 h before measurement of minimal and maximal fluorescence. Minimal fluorescence (*F*_o_) with all PSII reaction centers open was determined with modulated light that was sufficiently low to prevent significant variable fluorescence yield in the dark-adapted state (*F*_v_). Maximal fluorescence (*F*_m_) with all reaction centers closed was determined by irradiating a dark-adapted leaf for 0.8 s with saturating light of 8,000 μmol m^−2^ s^−1^. Then, the leaf was illuminated with actinic light of 500 μmol m^−2^ s^−1^. Steady-state fluorescence (*F*_s_) was recorded when the leaf attained steady-state photosynthesis. A second pulse of 0.8 s of saturating light of 8,000 μmol m^−2^ s^−1^ was applied to determine the *F*_m_ in the light-adapted state ($F_{\text{m}}\prime$) (Sui, 2015). The maximal photochemical efficiency (*F*_v_/*F*_m_) of PSII was expressed as *F*_v_/*F*_m_ = (*F*_m_ − *F*_o_)/*F*_m_. The quantum yield of PSII electron transport was determined using the formula ΦPSII = ($F_{\text{m}}\prime$ − *F*_s_)/$F_{\text{m}}\prime$. Non-photochemical quenching (NPQ) was calculated as NPQ = *F*_m_/$F_{\text{m}}\prime$ − 1. The chlorophyll fluorescence of seedlings that underwent recovery treatment for 3 days was measured in the same manner.

### Measurement of PSI capacity

The capacity of PSI was determined by measuring the absorbance at 820 nm (Δ*I*/*I*_o_) using a Plant Efficiency Analyser (Hansatech, King's Lynn, UK) after the leaves were treated in the dark for 30 min (Schansker et al., 2003; Qin et al., 2011). The first reliable measurement point for fluorescence change was at 20 μs, whereas the first measurement point for transmission change was at 400 μs. The transmission measurement point was 100 μs. Light intensity was 3,000 μmol m^−2^ s^−1^ photon flux density. The far-red source was a QDDH73520 light-emitting diode (LED; Quantum Devices Inc., Barneveld, WI, USA) filtered at 720 ± 5 nm. The modulated (33.3 kHz) far-red measuring light was provided by an OD820 LED (Opto Diode Corp., Newbury Park, CA, USA) filtered at 830 ± 20 nm.

### Antioxidant enzyme activity

Ascorbate peroxidase (APX) activity was determined based on the decrease in absorbance at 290 nm. The reaction mixture contained 50 mM potassium phosphate buffer (pH 7.0), 0.5 mM ascorbate, 0.2 mM H_2_O_2_, and a suitable volume of enzyme extract (Jimenez et al., 1997). Superoxide dismutase (SOD) activity was determined in accordance with the method described by Giannopolitis and Ries (1977). The leaves (same position, 0.5 g) were homogenized using a pre-cooled mortar and pestle in 5 mL reaction mixture containing 50 mM potassium phosphate buffer (pH 7.0) and 1% polyvinylpyrrolidone. The homogenate was centrifuged at 12,000 rpm for 10 min to collect the supernatant.

### Superoxide and hydrogen peroxide assays

Superoxide anion (${\text{O}}_{2}^{-•}$) content was assayed in accordance with the method described by Wang and Luo (1990). Fresh leaves with the midrib excised were ground in 0.05 M phosphate buffer (pH 7.8) in an ice bath. The homogenate was centrifuged (5,000 × g) at 4°C for 10 min to collect the supernatant. The supernatant was mixed with phosphate buffer (pH 7.8) and 10 mM hydroxyl ammonium chloride and incubated at 25°C for 20 min. After addition of 17 mM *p*-aminobenzene sulfonic acid and 7 mM α-naphthylamine, the mixture was incubated at 25°C for 20 min. Finally, ethyl ether was added to the mixture, followed by centrifugation at 1,500 × g for 5 min. Absorbance at 530 nm was determined in the water phase. The ${\text{O}}_{2}^{-•}$ generation was expressed as the content per gram of fresh leaf mass (Sui et al., 2007).

Hydrogen peroxide (H_2_O_2_) content was determined using the method described by Sairam and Srivastava (2002). The concentration of H_2_O_2_ was estimated by measuring the absorbance of the titanium–hydroperoxide complex, followed by mapping to a standard curve plotted with H_2_O_2_ standards (Sui et al., 2007).

### Measurement of the relative electric conductivity

The relative electric conductivity (REC) was assayed in accordance with the method described by Guo et al. (2015), using the two-leaf stage seedlings, which were treated for 4 days by various NaCl concentrations (50, 100, 150, 200, 250, 300, and 400 mM). 30 disks from the first three true leaves were placed into a tube containing 10 ml of distilled water. The tube was then shaken for 12 h at 180 rpm and the initial electric conductivity of the solution (S1) was measured. Then tubes were kept in the boiling water for 10 min and cooled down to room temperature. The final electric conductivity (S2) was then measured. The relative electric conductivity (REC) was calculated as follows: REC (%) = S1/S2 × 100.

### RNA isolation and illumina sequencing

Total RNA was extracted from leaf tissue sampled from seedlings in the S4, R3, and CK treatments using the RNA plant Plus reagent (DP437, Tiangen, Beijing, China) in accordance with the manufacturer's instructions (Sui et al., 2015; Yuan et al., 2015; Yang et al., 2017). RNA extracts were quantified using a Nanodrop ND-1000 spectrophotometer (Thermo Fisher Scientific, Wilmington, DE, USA). The extracts were electrophoresed in 1% agarose gel buffered with Tris–acetate–EDTA to determine the integrity of the RNA. RNA libraries were constructed following the High Throughput Illumina Strand-Specific RNA Sequencing Library protocol (Zhong et al., 2011). Briefly, mRNA was purified from 5 μg total RNA using oligo (dT) magnetic beads. The purified mRNA was fragmented into small pieces using a fragmentation buffer. Using these short fragments as templates, first-strand cDNA was synthesized using reverse transcriptase and random hexamer primers. Second-strand cDNA synthesis was followed using DNA polymerase I and RNase H.

### Gene annotation and classification

To obtain sequences of high similarity, unigene sequences were first aligned to sequences lodged in the protein databases NR (Deng et al., 2006), KEGG (Kanehisa et al., 2004), Swiss-Prot (Apweiler et al., 2004), and COG (Tatusov et al., 2000) (*e*-value < 0.00001) using the blastx tool, and the nucleotide database NT (*e-*value < 0.00001) using blastn. The KEGG database enables analysis of the gene product during the metabolism process and the related gene function in the cellular processes. KEGG pathways were assigned to the assembled sequences using the online KEGG web server (http://www.genome.jp/kegg/).

### Mapping and detection of differentially expressed genes

Six tag libraries were constructed for both shoots and roots sampled for each of the CK, S4, and R3 treatments. Reads per kilobase per million (RPKM) values were calculated using an in-house script based on the count table of Cuffdiffs output (http://cole-trapnell-lab.github.io/cufflinks/). The mapping and detection of DEGs were performed as described in a previous study (Sui et al., 2015). The DEGs with a fold change of ≥2 and divergence probability ≥0.8 between different samples were identified using the Noiseq method (Tarazona et al., 2011).

### Quantitative real-time PCR analysis

Quantitative real-time RT-PCR (qRT-PCR) analysis was performed to validate the RNA-seq results. Twelve DEGs were randomly selected for qRT-PCR. All primers were designed using the Beacon Designer software (version 7.9) (Additional file: Table S1). The housekeeping gene *Tubulin* (GenBank accession GO264294) was used as an internal standard (Chi et al., 2012). Total RNA (1 μg) was used in a 20 μl reaction volume for reverse transcription using the ReverTra Ace® qPCR RT Kit (Toyobo, Japan) in accordance with the manufacturer's instructions. Real-time PCR was performed in a 20 μl reaction volume containing 10 μl SYBR® Premix Ex Taq, 0.5 μl primer pairs (*tubulin* and target gene), and 2 μl (50 ng μl^−1^) cDNA. Real-time PCR was carried out on a real-time quantitative PCR instrument (LightCycler® 96, Roche Diagnostics, Mannheim, Germany). The reaction conditions were as follows: 95°C for 5 min, followed by 40 cycles of 95°C for 15 s, 55°C for 20 s, and 72°C for 20 s. Three biological replicates were included in each experiment.

### Measurement of ω-3 fatty acid desaturase activity

The ω-3 fatty acid desaturase protein was extracted using the following steps. Leaf samples (0.2 g) were frozen in liquid nitrogen, then ground in 2 ml extraction buffer (100 mM phosphate buffer, pH 7.2, containing 0.1 mM EDTA and 2 mM ascorbic acid). The homogenate was centrifuged at 10,000 rpm for 15 min, and the supernatant was used to measure enzyme activity. The activity of ω-3 fatty acid desaturase was measured with a ω-3 fatty acid desaturase activity kit using the double antibody sandwich method (Jie Shi Kang Biological Technology Co., Ltd, Qingdao, China).

### Statistical analysis

The software package SPSS 16.0 (SPSS, Chicago, IL, USA) was used for all statistical analyses following the method described in a previous study (Cheng et al., 2014). All data are presented as the mean (±SD) of five replicates (*n* = 5). Comparisons among multiple groups were performed using Duncan's multiple range test. Probability values *P* < 0.05 were considered statistically significant.

## Results

### Salt stress inhibited plant growth

**Figure 1:**
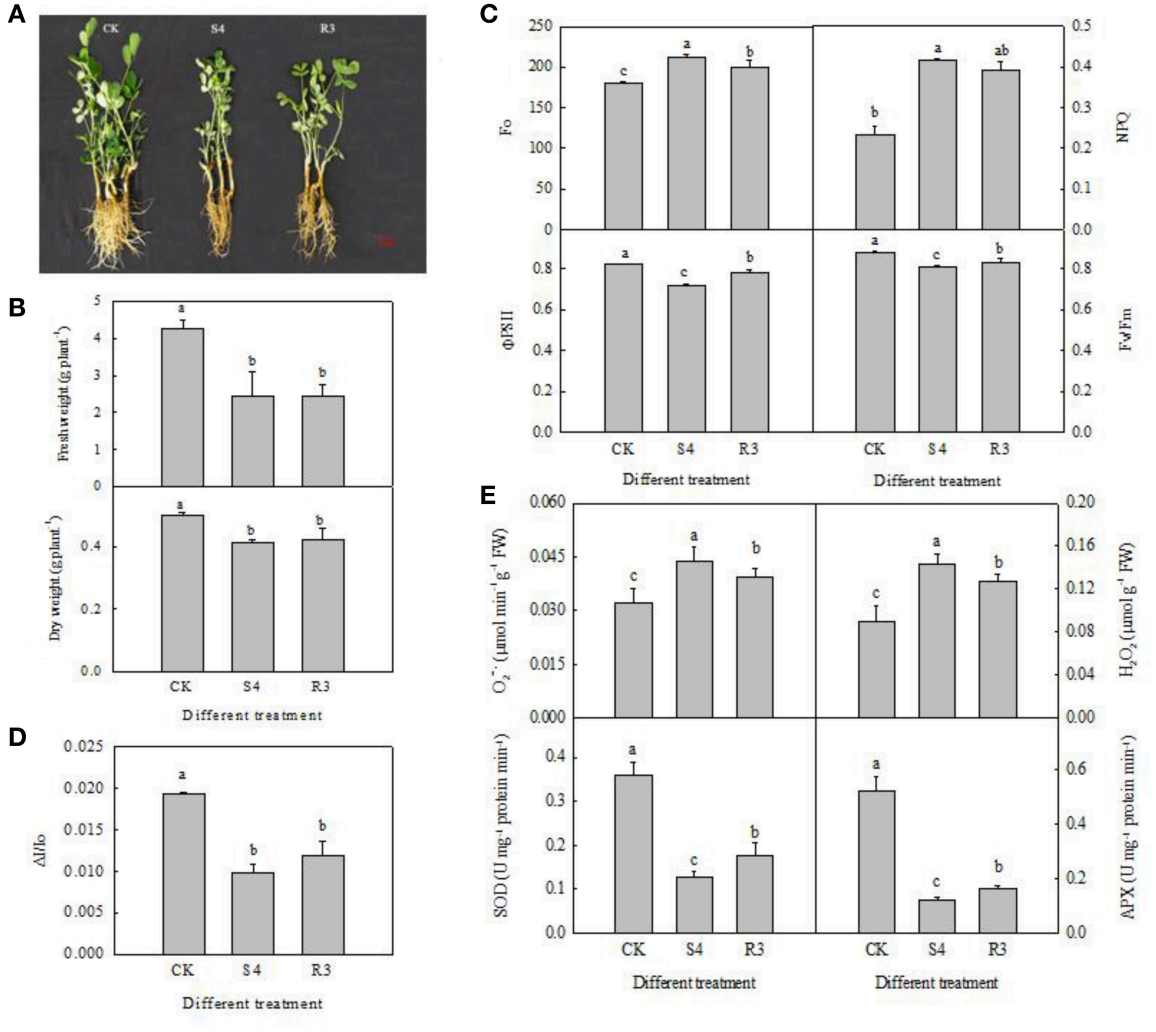
Growth of peanut seedlings in different treatments of CK, S4 and R3 **(A)**; seedling fresh weight and dry weight **(B)**; *F*_o_, NPQ, ΦPSII and *F*_v_/*F*_m_ **(C)**; changes in Δ*I*/*I*_o_ **(D)**; APX and SOD activity, and contents of H_2_O_2_ and ${\text{O}}_{2}^{-•}$ in the leaf **(E)**. Data are presented as the mean (±SD) of five replicates (*n* = 5). For each column, different lower-case letters indicate a significant difference at *P* < 0.05.

We analyzed the relative electric conductivity (REC) using the two-leaf stage seedlings, which were treated for 4 days by different NaCl concentrations of 50, 100, 150, 200, 250, 300, and 400 mM. Result showed that there was no significantly difference before 150 mM NaCl treatment. REC increased significantly at 250 mM NaCl treatment. According to these data, 250 mM NaCl for 4 days (S4) were used for further experiments (Figure S2). Treatment with 250 mM NaCl for 4 days significantly inhibited growth of the peanut seedlings (Figure 1A). Furthermore, the seedling fresh and dry weights decreased by 42.9 and 17.6%, respectively, under the S4 condition (Figure 1B). No significant differences in seedling fresh and dry weights were observed between the R3 and S4 conditions (Figure 1A).

### Salt stress induced changes in fatty acid composition in membrane lipids

Treatment with 250 mM NaCl for 4 days increased the contents of the saturated fatty acids palmitic acid (16:0) and stearic acid (18:0) and the unsaturated fatty acid 18:2, whereas the contents of palmitoleic acid (16:1) and 18:3 decreased (Table 1). Under the S4 condition, compared with CK, the 16:1 content decreased by 38.3% and the 18:2 content increased by 13.2%. In addition, the double bond index (DBI) and 18:3 content after S4 treatment decreased by 30.2 and 42.5%, respectively. Furthermore, the 16:1 content decreased by 8.5% and the 18:2 content increased by 5.7% after R3 treatment compared with those observed after S4 treatment. Moreover, the DBI and 18:3 content increased by 18.1 and 27.6%, respectively, after R3 treatment compared with those observed after S4 treatment.

**Table 1:** Constituent fatty acids of total lipids in peanut leaves.

**Fatty acid**	**CK**	**S4**	**R3**
16:0	64.32 ± 1.35^b^	68.29 ± 0.42^a^	67.37 ± 0.36^a^
16:1	2.09 ± 0.27^a^	1.29 ± 0.14^b^	1.18 ± 0.06^bc^
18:0	10.61 ± 1.03^c^	12.35 ± 0.73^a^	11.12 ± 0.24^b^
18:1	8.74 ± 0.12^a^	8.66 ± 0.14^a^	8.87 ± 0.26^a^
18:2	2.19 ± 0.27^bc^	2.48 ± 0.18^ab^	2.62 ± 0.09^a^
18:3	12.05 ± 0.22^a^	6.93 ± 0.09^c^	8.84 ± 0.03^b^
DBI	49.27 ± 1.24^a^	34.41 ± 0.15^c^	40.63 ± 0.21^b^

*Each point represents the mean ± SD of five measurements on each of five plants. Different letters a, b, and c indicate a significant difference at P < 0.05*.

### Salt stress affected PSII capacity

To evaluate the effect of the salt and recovery treatments on PSII capacity, we measured the *F*_o_, NPQ, ΦPSII and *F*_v_/*F*_m_ of peanut leaves in each treatment. *F*_o_ reflects the minimum fluorescence intensity after dark adaptation. The direction of *F*_o_ depends on the dominant factor for PSII inactivation, or damage and energy dissipation. Under the S4 condition, *F*_o_ and NPQ increased by 17.7 and 79.6%, respectively. Although NPQ decreased by 5.5% under the R3 condition, compared with that observed under the S4 condition, the difference was not statistically significant (Figure 1C). Under the S4 condition, ΦPSII and *F*_v_/*F*_m_ decreased by 12.7 and 8.5%, respectively, compared with the control. However, ΦPSII and *F*_v_/*F*_m_ increased by 8.9 and 3.0%, respectively, under the R3 condition compared with those observed under the S4 condition (Figure 1C).

### PSI oxidoreductive activity decreased under salt stress

Treatment with 250 mM NaCl decreased PSI oxidoreductive activity (Δ*I*/*I*_0_) in the leaf by 49.3% (Figure 1D). However, no significant differences in PSI oxidoreductive activity were observed between the R3 and S4 conditions (Figure 1D). Therefore, PSI oxidoreductive activity was reduced under salt stress but was not reversed by recovery.

### Salt stress decreased antioxidant enzyme activity and increased the ${\text{O}}_{2}^{-•}$ and H_2_O_2_ contents

Compared with the control, under the S4 and R3 conditions SOD activity decreased by 64.4 and 50.8%, respectively (Figure 1E), and APX activity decreased by 76.3 and 68.5%, respectively (Figure 1E). However, the activities of SOD and APX increased by 38.4 and 33.0%, respectively, under the R3 condition compared with those observed under the S4 condition. The contents of ${\text{O}}_{2}^{-•}$ and H_2_O_2_ exhibited a negative correlation with APX and SOD activity under the S4 and R3 conditions. The contents of ${\text{O}}_{2}^{-•}$ and H_2_O_2_ increased by 35.4 and 61.1%, respectively, after treatment with 250 mM NaCl for 4 days (Figure 1E). Under the R3 condition, ${\text{O}}_{2}^{-•}$ and H_2_O_2_ content decreased by 9.6 and 11.3%, respectively, compared with those observed under the S4 condition. Hence, salt stress inhibited antioxidant enzyme activity and increased the contents of ${\text{O}}_{2}^{-•}$ and H_2_O_2_, but recovery treatment reversed these changes.

### Identification of DEGs in response to salt stress

**Figure 2:**
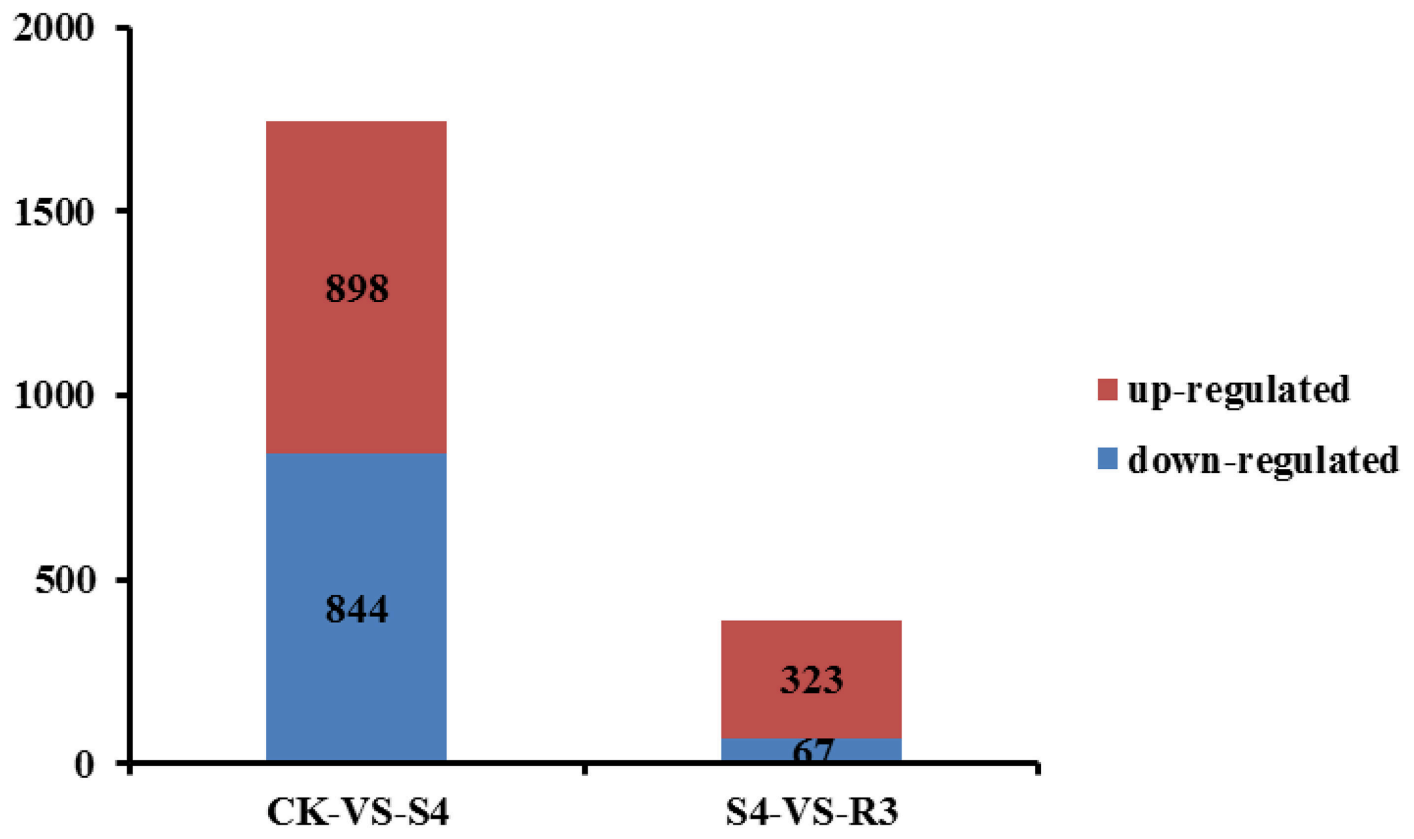
Numbers of differentially expressed genes in leaves of peanut seedlings in comparisons between treatments CK vs. S4, and S4 vs. R3. Unigenes were first aligned using the BlastX tool to sequences in protein databases (*e*-value < 0.00001).

**Figure 3:**
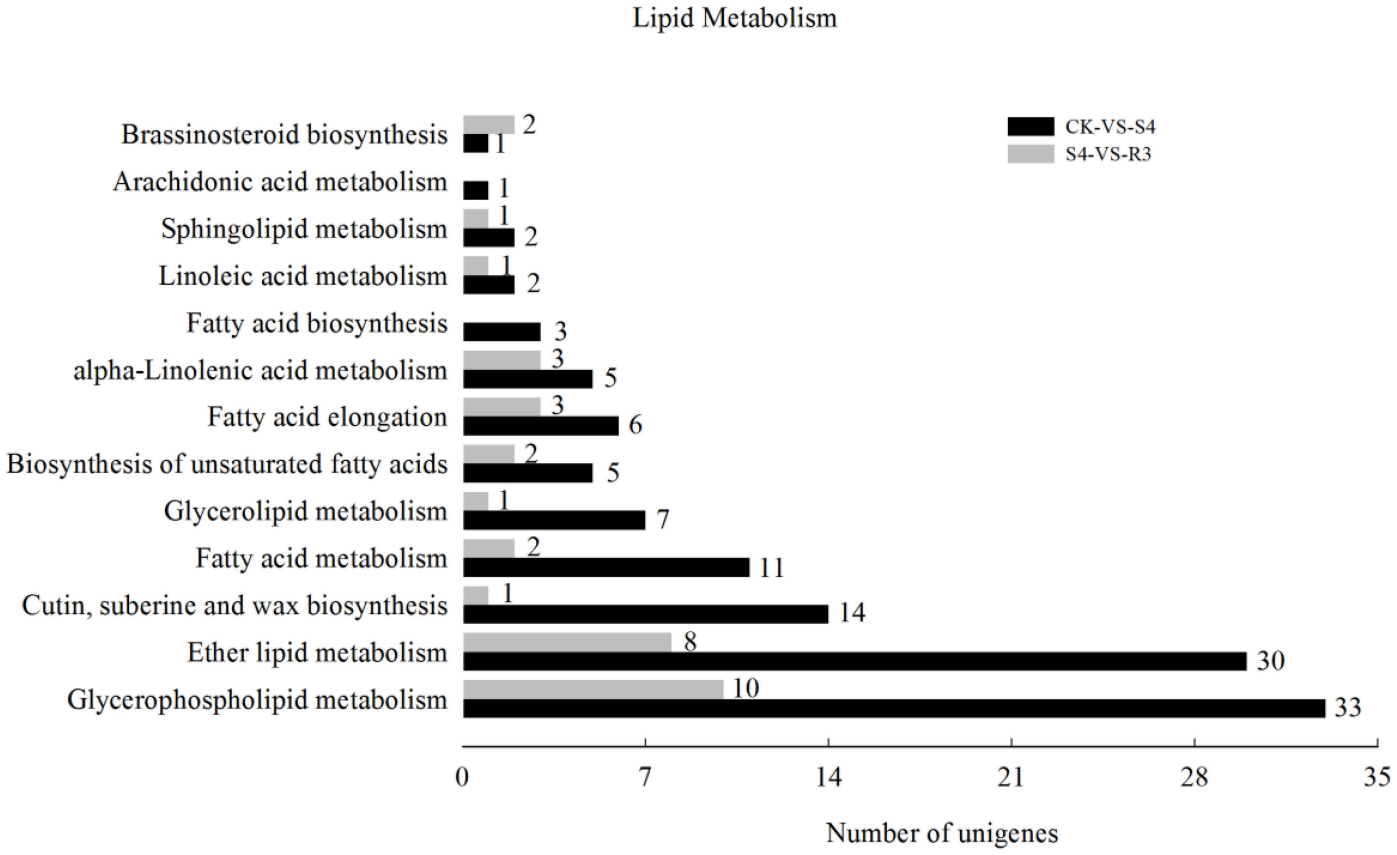
Numbers of differentially expressed genes associated with lipid metabolism in comparisons between treatments CK vs. S4, and S4 vs. R3. Unigenes were first aligned using the BlastX tool to sequences in protein databases (*e-* value < 0.00001).

To investigate the molecular mechanisms of salt tolerance in peanut seedlings and the recovery condition, RNA samples were extracted from leaves of the control (without NaCl treatment), salt-stress treatment (S4), and recovery treatment (R3) to prepare libraries for RNA-seq analysis. After stringent quality assessment and data filtering, 263.13 million clean reads were obtained (Table S2). Pearson correlation coefficients with a divergence probability ≥0.8 between RNA-seq samples were evaluated based on the square of the Pearson's correlation coefficient. The *R*^2^ values were all higher than 0.92, which indicated high repeatability (Figure S4). Compared with the control, 1,742 genes were differentially expressed under the S4 condition. Among these DEGs, 898 genes were up-regulated and 844 genes were down-regulated in the leaf under salt stress. Furthermore, 390 genes were differentially expressed under the R3 condition compared with the S4 condition. Among these DEGs, 323 genes were up-regulated and 67 genes were down-regulated in the leaf (Figure 2). A total of 120 genes differentially expressed between the S4 and CK conditions were mapped to 13 pathways associated with lipid metabolism (Figure 3). In addition, 34 genes differentially expressed between the S4 and R3 conditions were mapped to 11 pathways (Figure 3).

### Genes associated with lipid metabolism were differentially expressed under the salt stress and recovery

As important components of cell membrane phospholipids, polyunsaturated fatty acids determine the fluidity and deformability of the cell membrane by maintaining the structure and function of the cell membrane. Previous studies have observed that lipids might play important roles in protecting the plant against salt stress (Huflejt et al., 1990; Khamutov et al., 1990; Ritter and Yopp, 1993; Sui et al., 2017). In the present study, 26 DEGs were mapped to five lipid metabolism-related pathways, and 11 DEGs were mapped to the fatty acid metabolism pathway (Table 2). Five genes were categorized as long-chain acyl-CoA synthetase. Among these five genes, four genes were down-regulated and one gene was up-regulated under the S4 condition. Gene CL639.Contig19, which was down-regulated under the S4 condition and up-regulated under the R3 condition, was categorized as oxidoreductase. Three genes that encoded alcohol dehydrogenase were up-regulated under the S4 condition. The DEGs that encoded the protein VITISV_013417 and MFP-a were up-regulated under the S4 condition, but no significant changes were observed under the R3 condition.

**Table 2:** DEGs mapped to KEGG pathways related to lipid metabolism.

**Gene ID**	**Anotation**	**S4-VS-CK**			**R3-VS-S4**		
		**Log_2_FC**	**Regulated**	**Probability**	**Log_2_FC**	**Regulated**	**Probability**
**(A) FATTY ACID METABOLISM 11 71**							
CL1692.Contig15	long chain acyl-CoA synthetase 2	3.00	Up	0.82	–	–	0.00
CL7059.Contig2	long chain acyl-CoA synthetase 1	−2.13	Down	0.83	–	–	0.00
CL3870.Contig1	long chain acyl-CoA synthetase 2	−3.02	Down	0.86	2.18	Up	0.80
CL1692.Contig4	long chain acyl-CoA synthetase 2	−9.14	Down	0.88	–		0.00
CL1692.Contig12	long chain acyl-CoA synthetase 2	−9.09	Down	0.87	–		0.00
CL639.Contig19	oxidoreductase activity	−3.15	Down	0.88	2.18	Up	0.84
CL2565.Contig1	alcohol dehydrogenase, partial	4.63	Up	0.92	–	–	0.00
Unigene13905	alcohol dehydrogenase	4.07	Up	0.91	–		0.00
Unigene13904	alcohol dehydrogenase (NAD) activity	4.47	Up	0.91	–		0.00
Unigene4960	hypothetical protein VITISV_013417	4.98	Up	0.88	–		0.00
CL6052.Contig9	glyoxysomal fatty acid beta-oxidation multifunctional protein MFP-a	2.55	Up	0.86	–		0.00
**(B) BIOSYNTHESIS OF UNSATURATED FATTY ACIDS 5**							
CL8534.Contig6	ω-3 fatty acid desaturase	−5.11	Down	0.92	4.14	Up	0.88
CL8534.Contig7	ω-3fatty acid desaturase	−3.02	Down	0.88	1.98	Up	0.83
Unigene10822	short-chain type dehydrogenase	2.31	Up	0.85	–	–	0.00
Unigene3398	elongation of fatty acids protein 1	−3.42	Down	0.86	–	–	0.00
Unigene19394	peroxisomal 3-ketoacyl-CoA thiolase	1.84	Up	0.81	–	–	0.00
**(C) ALPHA-LINOLENIC ACID METABOLISM 5 592**							
Unigene5941	4-coumarate-CoA ligase	−3.28	Down	0.83	–	–	0.00
CL7132.Contig3	7-methylxanthosine synthase 1	7.33	Up	0.86	–	–	0.00
CL3328.Contig1	12-oxophytodienoate reductase 2	–	–	0.00	−2.34	Down	0.84
Unigene27104	12-oxo-phytodienoate reductase 2	–	–	0.00	2.02	Up	0.81
Unigene27102	12-oxo-phytodienoate reductase 2	–	–	0.00	2.78	Up	0.88
**(D) FATTY ACID BIOSYNTHESIS 3 61**							
CL415.Contig3	serine-type endopeptidase activity	−4.37	Down	0.86	–	–	0.00
Unigene10129	glucose 1-dehydrogenase [NAD(P)] activity	3.94	Up	0.84	–	–	0.00
CL415.Contig2	serine-type endopeptidase activity	−2.05	Down	0.82	–	–	0.00
**(E) LINOLEIC ACID METABOLISM 2 591**							
Unigene11867	WW domain-containing oxidoreductase	−3.13	Down	0.86	–	–	0.00
CL1272.Contig5	Retinol dehydrogenase	−2.87	Down	0.87	2.33	Up	0.86

Five DEGs were mapped to the biosynthesis of the unsaturated fatty acids pathway. Among these five DEGs, two genes (CL8534.Contig6 and CL8534.Contig7) were categorized as ω-3 fatty acid desaturase. These two genes were involved in the synthesis of 18:3 from 18:2 (Additional file: Figure S3). The expression of these two genes decreased under the S4 condition but increased under the R3 condition (Table 2). The unigene10822 and unigene19394, which encode short-chain-type dehydrogenase and 3-ketoacyl-CoA thiolase, were up-regulated under the S4 condition. The DEG that encoded the elongation of fatty acids protein was down-regulated under the S4 condition.

Five DEGs were mapped to the α-linolenic acid metabolism pathway (Table 2). A DEG that encoded 4-coumarate-CoA ligase was down-regulated under the S4 condition. CL7132.Contig3, which encoded 7-methylxanthosine synthase 1, was up-regulated under the S4 condition. Three DEGs that encoded 12-oxophytodienoate reductase 2 were differentially expressed under the R3 condition. Furthermore, the expression levels of unigene27104 and unigene27102 increased under the R3 condition, whereas the expression level of CL3328.Contig1 decreased (Table 2).

Three DEGs were mapped to the fatty acid biosynthesis pathway. Two DEGs that encoded serine-type endopeptidase were down-regulated under the S4 condition. The expression of unigene10129, which encoded glucose 1-dehydrogenase, was up-regulated under the S4 condition.

Two DEGs were mapped to the linoleic acid metabolism pathway. The unigene11867, which encoded oxidoreductase, was down-regulated under the S4 condition. The expression level of CL1272.Contig5, which encoded retinol dehydrogenase, also decreased under the S4 condition, but increased under the R3 condition (Table 2).

### High consistency between RNA-seq and qRT-PCR

**Figure 4:**
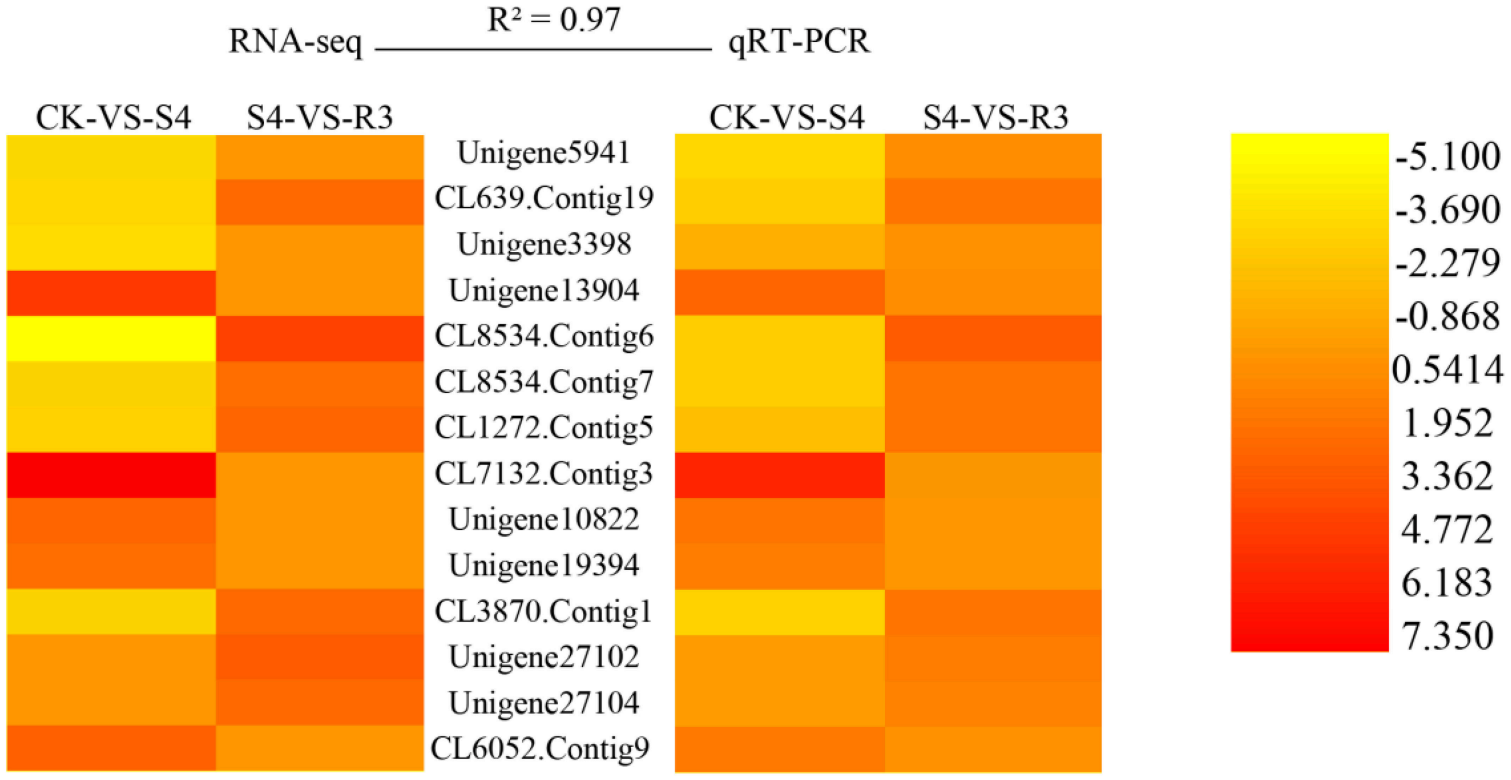
Validation of RNA-seq data by quantitative real-time PCR (qRT-PCR). Twelve DEGs were selected for the qRT-PCR analysis. *R*^2^ represents the correlation coefficient value between the two platforms. The values for the scale bar represent log_2_FC values for RNA-seq and −ΔΔCt for qRT-PCR, which were used to evaluate the correlation (*R*^2^). Primers used are listed in Table S1.

To validate the RNA-seq data, qRT-PCR analysis were performed on 14 randomly selected DEGs. As shown in Figure 4, the high reliability of the RNA-seq data was confirmed by the high correlation value (*R*^2^ = 0.97).

### Expression level of CL8534.Contig6 decreased under salt stress and increased under recovery

**Figure 5:**
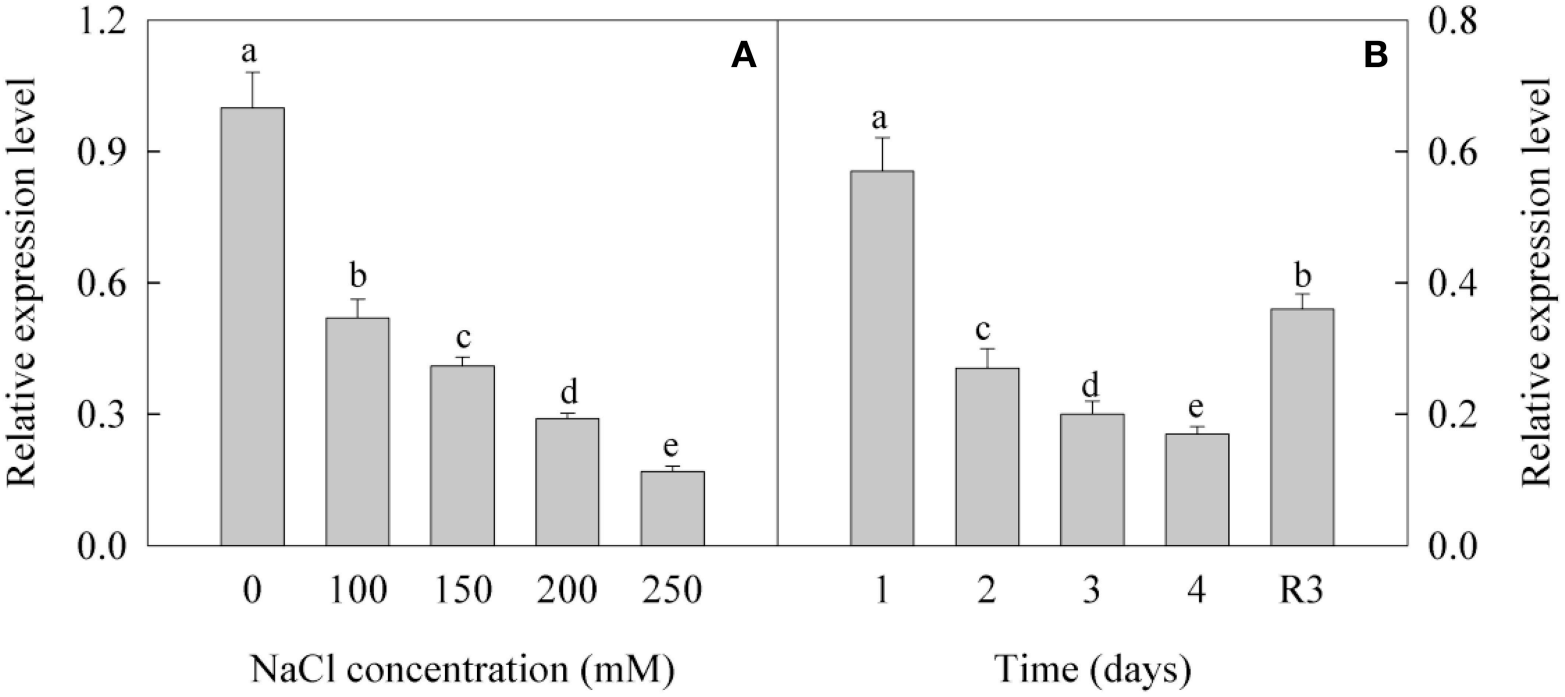
Expression level of CL8534.Contig6 in response to 0, 100, 150, 200, and 250 mM NaCl treatment for 4 days **(A)** and recovery for 3 days **(B)**. Data are means (±SD) of five replicates (*n* = 5). For each column, different lower-case letters indicate a significant difference at *P* < 0.05.

The expression of CL8534.Contig6 was down-regulated under treatment with different concentrations of NaCl. After treatment with 100, 150, 200, and 250 mM NaCl, the expression level of CL8534.Contig6 decreased by 48.1, 58.9, 71.3, and 82.9%, respectively (Figure 5A). Under treatment with 250 mM NaCl, the expression level of CL8534.Contig6 gradually decreased with prolonged duration of exposure. Under the R3 condition, the expression level of CL8534.Contig6 increased by 111.8% compared with the level measured after treatment with 250 mM NaCl for 4 days (Figure 5B).

### The ω-3 fatty acid desaturase activity decreased under salt stress and increased under recovery

**Figure 6:**
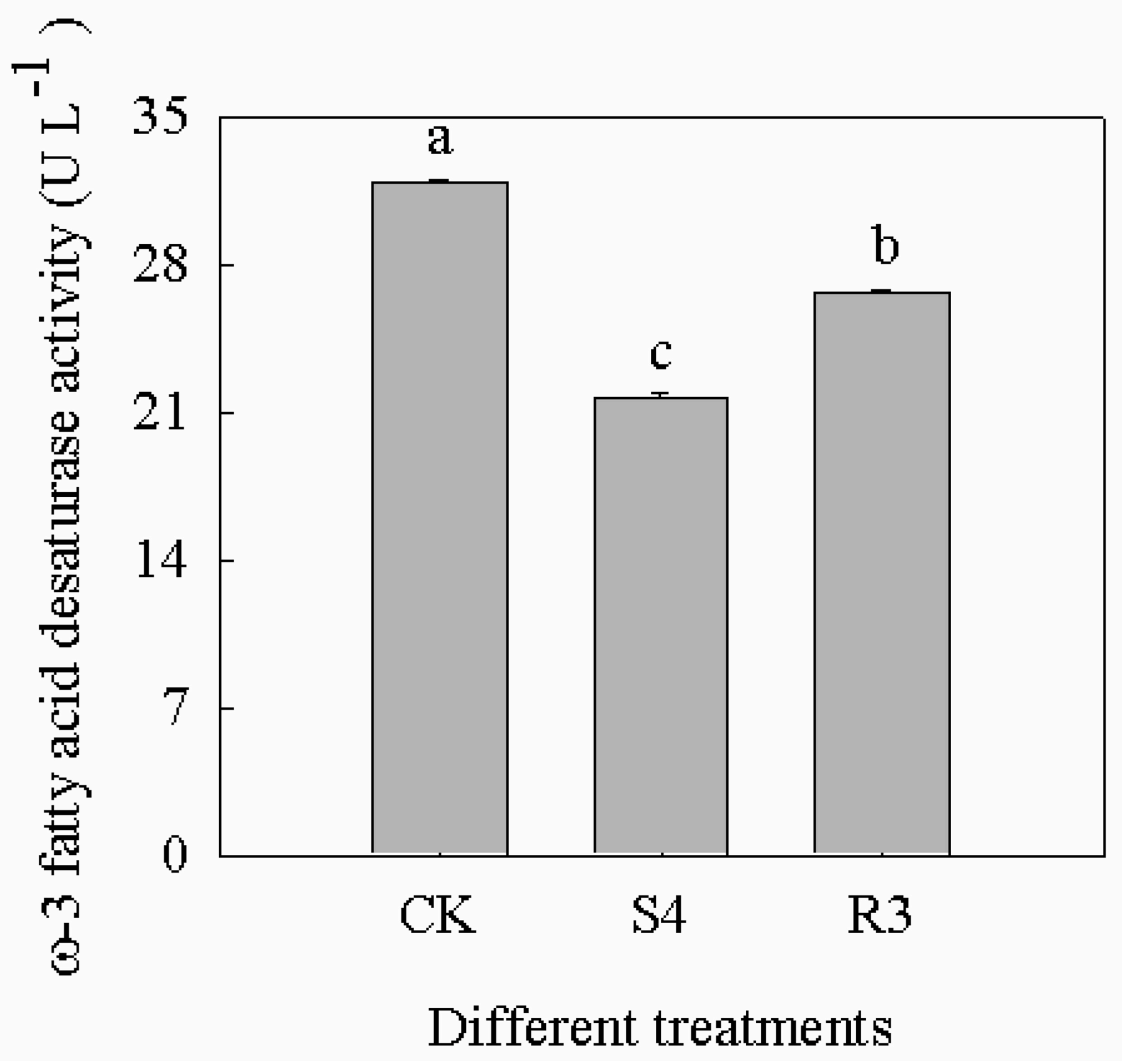
Activity of ω-3 fatty acid desaturase at different treatments of CK, S4 and R3. Data are means (±SD) of five replicates (*n* = 5). For each column, different lower-case letters indicate a significant difference at *P* < 0.05.

Treatment with 250 mM NaCl decreased the activity of ω-3 fatty acid desaturase by 32.1%. Furthermore, the activity of ω-3 fatty acid desaturase increased by 23.0% under the R3 condition, compared with that observed under the S4 condition, but remained lower than that of the CK (Figure 6).

## Discussion

Salt stress can inhibit plant development. In the present study, 250 mM NaCl caused a dramatic decrease in leaf length, leaf number, FW, DW, and root length in peanut seedlings (Figures 1A,B). These physiological indicators were not reversed by recovery treatment, which indicated that the growth of peanut seedlings was severely inhibited by salt stress and was not recovered when transferred to standard (ST) conditions.

With a hydrophobic interior of lipids, cell membranes serve as a barrier to regulate the transportation of most ions and large molecules. As important components of cell membrane phospholipids, polyunsaturated fatty acids determine the fluidity and deformability of the cell membrane. Polyunsaturated fatty acids play a key role in maintaining the structure and function of the cell membrane (Mikami and Murata, 2003). Our previous studies revealed that increased unsaturated fatty acid content in membrane lipids of *Suaeda salsa* enhanced the salt tolerance of PSII (Sui et al., 2010, 2017; Cheng et al., 2014). In addition, higher levels of phosphatidylglycerol (PG), sulfoquinovosyldiacylglycerol (SQDG) and DBI, and a higher ratio of digalactosyldiacylglycerol/monogalactosyldiacylglycerol (DGDG/MGDG) were observed in young *S. salsa* seedlings treated with 500 mM NaCl compared with those treated with 1 mM NaCl (Zhou et al., 2016). Mutations of the cyanobacteria *Synechocystis* lead to reduced polyunsaturated fatty acid content and increased susceptibility to low temperature and salt (Tasaka et al., 1996; Allakhverdiev et al., 1999). In the present study, the constituent fatty acids of total lipids in peanut leaves were measured. The results revealed that DBI, as well as 16:1 and 18:3 contents, significantly decreased under the S4 condition, whereas 18:2 content slightly increased, which indicated that 18:3 content was significantly affected by treatment with 250 mM NaCl. Furthermore, ΦPSII, *F*_v_/*F*_m_ and PSI oxidoreductive activity (Δ*I*/*I*_o_) decreased under the S4 condition, whereas *F*_o_ and NPQ increased (Figure 1C), which indicated that PSII and PSI were both affected by the high concentration of salt. Changes in *F*_o_ depend on the dominant factor in PSII inactivation or damage and energy dissipation. The remarkable increase in *F*_o_ and NPQ in the present study might be an indication of PSII inactivation or damage by salt stress. The present data revealed that the reduction in unsaturated fatty acid content led to the photoinhibition of PSII and PSI under salt stress, which was consistent with the result in *Synechococcus* that the combination of light and the fatty acid unsaturation is shown to be the most effective way to protect the photosynthetic machinery (Allakhverdiev et al., 2001). This photoinhibition would inevitably lead to the production of reactive oxygen species (ROS) in cells and cause oxidative damage to chloroplasts (Chen et al., 2004). Detoxification of ROS is essential for metabolism under non-stress and stress conditions. Enzymes such as SOD, APX, and peroxidase play important roles in scavenging ROS (Apel and Hirt, 2004; Mittler et al., 2004; Dietz et al., 2006; Thakur et al., 2017). These mechanisms have been observed in almost all cellular compartments, which demonstrate the importance of ROS detoxification for cellular survival (Mittler et al., 2004; Vuleta et al., 2016). SOD is the key enzyme to scavenge ${\text{O}}_{2}^{-•}$ in chloroplasts. In the present study, the activity of SOD significantly decreased in response to salt stress, whereas the ${\text{O}}_{2}^{-•}$ content increased (Figure 1E). APT, as an antioxidant, can detoxify H_2_O_2_ into water and molecular oxygen (Huang et al., 2005; Guo et al., 2017). In the present study, the activity of APX decreased under the S4 condition, which in turn led to an increase in H_2_O_2_ content (Figure 1E). These data suggest that treatment with 250 mM NaCl decreases the activity of antioxidant enzymes and the ROS cannot be scavenged effectively, which induces the accumulation of ROS.

To further investigate the salt response in peanut seedlings, transcriptomic analysis was performed. Pathway analysis is an effective method to characterize “gene networks” under salt stress. A total of 1742 genes differentially expressed between the non-stress and S4 conditions, and 390 genes differentially expressed between the S4 and R3 conditions were identified. Among these DEGs, 27 DEGs were mapped to five lipid metabolism-related pathways (Table 2). In the fatty acid metabolism pathway, four long-chain acyl-CoA synthetases (LACS, EC .3) that encoded DEGs were down-regulated, whereas one DEG was up-regulated under the S4 condition (Table 2). LACS occupy a critical position in the biosynthetic pathways of almost all fatty acid-derived molecules. LACS esterifies free fatty acids to acyl-CoAs, which is a key activation step necessary for the utilization of fatty acids by most lipid metabolic enzymes. Furthermore, LACS plays a primary role in the synthesis of acyl-CoA molecules used as substrates for phospholipid and TAG biosynthesis (Klett et al., 2017; Teodoro et al., 2017). The acyl-CoAs are utilized by acyltransferases, which catalyze the successive acylations of glycerol-3-phosphate. The acylglycerol intermediates that are ultimately formed are converted into the suite of phospholipids necessary for membrane biosynthesis in all tissues of the plant (Shockey and Fulda, 2002). It appears that salt stress can lead to down-regulation of DEGs that encode LACS. The decreased expression of LACS genes decreases the content of phospholipids, and might affect cell membrane structure and stability. In the biosynthesis of unsaturated fatty acids, two DEGs (CL8534.Contig6 and CL8534.Contig7) that encoded ω-3 fatty acid desaturase were down-regulated under the S4 condition and up-regulated under the R3 condition. Changes in trienoic fatty acids caused by modulated ω-3 fatty acid desaturase activities are conducive for plant metabolic adaptation to environmental stresses, such as salinity stress, temperature stress and drought stress (Liu et al., 2006; Yu et al., 2009; Menard et al., 2017). The *FAD7*/*FAD8* double mutant of Arabidopsis, which is deficient in 18:3 content, is more susceptible to chilling stress (Gibson et al., 1994; McConn et al., 1994). Under high temperature, leaves of the *fad3-fad7-fad8* triple mutant of Arabidopsis would accumulate high concentrations of C16:0 and exhibit a decrease in C16:2 desaturated fatty acids, whereas no such changes in the synthesis of C18:3 fatty acids would be observed in wild types (Manan et al., 2017). The expression of the modified sesame ω-3 desaturase (fad7) increases the α-linolenic acid content in the range of 4.78–6.77% in the seeds of transgenic tobacco plants with concomitant decrease in linoleic acid content (Bhunia et al., 2016). The present results revealed that with the down-regulation of two DEGs (CL8534.Contig6 and CL8534.Contig7), ω-3 fatty acid desaturase activity decreased under the S4 condition. The reduction in ω-3 fatty acid desaturase activity inhibited 18:3 synthesis, leading to the increase in 18:2 content (Table 1). Compared with that under the S4 condition, the expression of CL8534.Contig6 and ω-3 fatty acid desaturase activity increased under the R3 condition, and remained lower than those under normal conditions (Figures 4, 5). These results indicate that treatment with 250 mM NaCl decreases the activity of ω-3 fatty acid desaturase and reduces unsaturated fatty acid content, which in turn inevitably affect membrane stability and fluidity.

In conclusion, the present results reveal that the expression of fatty acid desaturase genes, ω-3 fatty acid desaturase activity and unsaturated fatty acid content decrease under salt stress. The photosystem and enzymes located on the membrane were also affected and resulted in photoinhibition of PSII and PSI. Subsequently, ROS was induced by salt stress. The decreased activities of SOD and APX failed to effectively scavenge ROS. Hence, the ${\text{O}}_{2}^{-•}$ and H_2_O_2_ contents increased under the S4 condition. The RNA-seq data were consistent with analyses of physiological parameters. Further genetic and biochemical analysis would help in understanding the regulatory mechanism of unsaturated fatty acids in response to salt stress in peanut.

## Availability of supporting data

NA-seq data in this study have been deposited in the National Center for Biotechnology Information (NCBI) SRA database (accession number: PRJNA398720).

## Author contributions

NS and SL wrote this manuscript; SL, YW, and ZY performed experiments; SL, YW, and FW collected data and carried out all analyses; NS and SW conceptualized the idea and revised the manuscript.

### Conflict of interest statement

The authors declare that the research was conducted in the absence of any commercial or financial relationships that could be construed as a potential conflict of interest.
