# Supplementary material for: Transcriptomic and Physiological Evidence for the Relationship between Unsaturated Fatty Acid and Salt Stress in Peanut
Source: Front Plant Sci. 2018 Jan 22;9:7. doi: 10.3389/fpls.2018.00007 (PMC5786550; doi:10.3389/fpls.2018.00007)
Supplement: Supplementary file 1 [file Table1.docx]

Table S1 Primer pairs for real-time quantitative PCR

| Genes | Annotation | Sense primer ( 5’- 3’) | Antisense primer ( 5’- 3’) |
| --- | --- | --- | --- |
| Unigene5941 | 4-coumarate-CoA ligase activity | ATTCCATTTACTATCCCATTG | CTGAGACACTACTTAGAGG |
| CL639.Contig19 | oxidoreductase activity | GCTTGTTCTATATCTGTTA | TGTATCATTAGGTCTGTT |
| Unigene3398 | elongation of fatty acids protein 1-like | TTGGATGGAGTTGCTGAG | GCCTACATCTTCTACCTCTC |
| Unigene13904 | alcohol dehydrogenase (NAD) activity | GATTGAGTGAGTGATGAAT | TTGTATGAGAGGACTGTTA |
| CL8534.Contig6 | fatty acid desaturase | CGTGACTATGGTTGGATT | CTGCTTCAGTTGCTTCTA |
| CL8534.Contig7 | fatty acid desaturase | CGTGACTATGGTTGGATT | TTGGCTCTCGGTAATACT |
| CL1272.Contig5 | pollen tube growth;oxidation-reduction process | GCGAGTGATAGAGATTAG | GCTCCTCCTGTAATAATAC |
| CL7132.Contig3 | 7-methylxanthosine synthase 1 | ACCTGAAGAACATCCTAA | GCTAACAACTCATCACTT |
| Unigene10822 | short-chain type dehydrogenase, putative | AGGTCGAATCATAATGCT | GCTTTGGATGCTGAGTAT |
| Unigene19394 | peroxisomal 3-ketoacyl-CoA thiolase | GACAGTTAATAGGCAATG | TAGTTGACATAGATTCCA |
| CL3870.Contig1 | long chain acyl-CoA synthetase 2 | TCTCCTGTTGTTCCACTT | GTACATCCTGCTTCTCTTG |
| Unigene27102 | 12-oxophytodienoate reductase 2 | GCAGAGTATGTAGATTGTG | GTTATCATTCTCGGTTCAA |
| Unigene27104 | 12-oxophytodienoate reductase 2 | AATTGGAGAAGTTGGTAG | TTATACAAGGTTGAAGGAA |
| CL6052.Contig9 | glyoxysomal fatty acid beta-oxidation multifunctional protein MFP-a | ATGATGTTAAGGCTATTG | CTTCTACAGTATCAGTGA |
