# Supplementary material for: Transcriptomic and Physiological Evidence for the Relationship between Unsaturated Fatty Acid and Salt Stress in Peanut
Source: Front Plant Sci. 2018 Jan 22;9:7. doi: 10.3389/fpls.2018.00007 (PMC5786550; doi:10.3389/fpls.2018.00007)
Supplement: Supplementary file 2 [file Table2.doc]

**TABLE S2** Comparison results of each sample with the reference gene sequence

| **Sample** | **Total Reads** | **Total Base Pairs** | **Total Mapped Reads** | **Perfect Match** | **Mismatch** | **Unique Match** | **Multi-position Match** | **Total Unmapped Reads** |
| --- | --- | --- | --- | --- | --- | --- | --- | --- |
| CK4dR1 | 14633692  (100.00% ) | 1907701682  (100.00% ) | 14066859  (96.13% ) | 5452888  (37.26% ) | 8613971  (58.86% ) | 9517478  (65.04% ) | 4549381  (31.09% ) | 566832  (3.87% ) |
| CK4dR2 | 13438765  (100.00% ) | 1800651324  (100.00% ) | 13002994  (96.76% ) | 5828600  (43.37% ) | 7174394  (53.39% ) | 8678891  (64.58% ) | 4324103  (32.18% ) | 435770  (3.24% ) |
| CK4dR3 | 17681434  (100.00% ) | 2511764379  (100.00% ) | 17098170  (96.70% ) | 7512959  (42.49% ) | 9585211  (54.21% ) | 11678232  (66.05% ) | 5419938  (30.65% ) | 583263  (3.30% ) |
| CK4dS1 | 13007394  (100.00% ) | 1564508011  (100.00% ) | 12692923  (97.58% ) | 4910470  (37.75% ) | 7782453  (59.83% ) | 8527278  (65.56% ) | 4165645  (32.03% ) | 314470  (2.42% ) |
| CK4dS2 | 13576432  (100.00% ) | 1875261517  (100.00% ) | 13253023  (97.62% ) | 5643911  (41.57% ) | 7609112  (56.05% ) | 8708533  (64.14% ) | 4544490  (33.47% ) | 323408  (2.38% ) |
| CK4dS3 | 15682992  (100.00% ) | 2054335175  (100.00% ) | 15347648  (97.86% ) | 6700343  (42.72% ) | 8647305  (55.14% ) | 10232127  (65.24% ) | 5115521  (32.62% ) | 335343  (2.14% ) |
| NaCl4dR1 | 16232410  (100.00% ) | 2200538804  (100.00% ) | 14716210  (90.66% ) | 6251493  (38.51% ) | 8464717  (52.15% ) | 9971360  (61.43% ) | 4744850  (29.23% ) | 1516199  (9.34% ) |
| NaCl4dR2 | 13307491  (100.00% ) | 1934065137  (100.00% ) | 12586332  (94.58% ) | 5302486  (39.85% ) | 7283846  (54.73% ) | 8621450  (64.79% ) | 3964882  (29.79% ) | 721158  (5.42% ) |
| NaCl4dR3 | 15551635  (100.00% ) | 1941782639  (100.00% ) | 14077908  (90.52% ) | 6442218  (41.42% ) | 7635690  (49.10% ) | 9383249  (60.34% ) | 4694659  (30.19% ) | 1473726  (9.48% ) |
| NaCl4dS1 | 15644250  (100.00% ) | 2115788520  (100.00% ) | 15242324  (97.43% ) | 6548289  (41.86% ) | 8694035  (55.57% ) | 9960523  (63.67% ) | 5281801  (33.76% ) | 401925  (2.57% ) |
| NaCl4dS2 | 12957543  (100.00% ) | 1695052838  (100.00% ) | 12549571  (96.85% ) | 5207056  (40.19% ) | 7342515  (56.67% ) | 8377713  (64.66% ) | 4171858  (32.20% ) | 407971  (3.15% ) |
| NaCl4dS3 | 14770876  (100.00% ) | 2002307220  (100.00% ) | 14318380  (96.94% ) | 5706095  (38.63% ) | 8612285  (58.31% ) | 9559081  (64.72% ) | 4759299  (32.22% ) | 452495  (3.06% ) |
| recover3dR1 | 12403875  (100.00% ) | 1582402923  (100.00% ) | 11134344  (89.77% ) | 4247430  (34.24% ) | 6886914  (55.52% ) | 7550653  (60.87% ) | 3583691  (28.89% ) | 1269530  (10.23% ) |
| recover3dR2 | 14646728  (100.00% ) | 1915369819  (100.00% ) | 12875887  (87.91% ) | 4768324  (32.56% ) | 8107563  (55.35% ) | 8749527  (59.74% ) | 4126360  (28.17% ) | 1770840  (12.09% ) |
| recover3dR3 | 13773367  (100.00% ) | 1786517797  (100.00% ) | 11992858  (87.07% ) | 4483372  (32.55% ) | 7509486  (54.52% ) | 8223655  (59.71% ) | 3769203  (27.37% ) | 1780508  (12.93% ) |
| recover3dS1 | 16818205  (100.00% ) | 2214792202  (100.00% ) | 16418016  (97.62% ) | 7084772  (42.13% ) | 9333244  (55.49% ) | 10845902  (64.49% ) | 5572114  (33.13% ) | 400188  (2.38% ) |
| recover3dS2 | 13591411  (100.00% ) | 1780596176  (100.00% ) | 12436531  (91.50% ) | 5126293  (37.72% ) | 7310238  (53.79% ) | 8380663  (61.66% ) | 4055868  (29.84% ) | 1154879  (8.50% ) |
| recover3dS3 | 15409945  (100.00% ) | 2163673181  (100.00% ) | 15014511  (97.43% ) | 6276897  (40.73% ) | 8737614  (56.70% ) | 9978818  (64.76% ) | 5035693  (32.68% ) | 395433  (2.57% ) |
